# Estimation of daily selenium intake by 3- to 5-year-old Japanese children based on selenium excretion in 24-h urine samples

**DOI:** 10.1017/jns.2019.21

**Published:** 2019-07-24

**Authors:** Yoshitaka Nakamura, Michiko Fukushima, Seiko Hoshi, Amares Chatt, Takashi Sakata

**Affiliations:** 1 Food Science & Technology Research Laboratories, Meiji Co., Ltd., Hachiouji, Tokyo 192-0919, Japan; 2 Ishinomaki Senshu University, Ishinomaki, Miyagi 986-8580, Japan; 3 Shokei Gakuin University, Natori, Miyagi 981-1295, Japan; 4 Department of Chemistry, Dalhousie University, Halifax, NS, B3H 4J3, Canada

**Keywords:** Early childhood, Micronutrients, Selenium intake, Urine, AAS, atomic absorption spectrometry, ICP, inductively coupled plasma, INAA, instrumental neutron activation analysis, NAA, neutron activation analysis, NIST, United States National Institute of Standards and Technology, PC-INAA, pseudo-cyclic instrumental neutron activation analysis, SRM, standard reference material

## Abstract

To evaluate the daily Se intake of 3- to 5-year-old Japanese children, we used seventy-two urine samples collected from fifty-three children (twenty-seven male and twenty-six female) from two cities in Miyagi prefecture, Japan. For measuring low Se concentrations with high precision, accuracy and rapidity in the 24-h urine samples, we developed an instrumental neutron activation analysis (INAA) method, that is without any chemical separation, using the short-lived ^77m^Se (*t*_1/2_ = 17·4 s) nuclide. The estimated Se intake of the fifty-three children was 51·5 (sd 30·2) µg/d (geometric mean: 42·7 µg/d). Ten subjects (three male and seven female), successfully provided 24-h urine samples over two or three consecutive days; their Se intake was 37·4 (sd 5·9) µg/d. Based on the logarithmically transformed data of these ten subjects, the ratio of intra-/inter-individual variances of usual Se intake was 16·7 (28·0/1·7) and geometric mean was 27·7 µg/d. The 5th to 95th percentile of usual Se intake of these ten subjects was 17·5 to 40·4 µg/d, which ranged between the recommended dietary allowance and tolerable upper intake level of Se by the Dietary Reference Intakes for Japanese (2015).

It is critical that children consume appropriate amounts of essential micronutrients from food for their healthy growth and development. However, scientific research has not adequately investigated the intake of essential micronutrients during early childhood worldwide, especially in Japan. Se is one of the most important essential micronutrients for antioxidant systems and thyroid hormone metabolism; its physiological function is expressed in the form of Se-containing proteins, including glutathione peroxidase, iodothyronine deiodinase and thioredoxin-disulphide reductase^(1)^. A lack of Se causes leg muscle pain, dry and flaking skin, and cardiomyopathy^(2,3)^. However, excessive Se intake causes the hair and nails to become fragile and fall out, as well as gastrointestinal disorders and skin rash^(1,4)^. A balanced intake of Se from food is essential to avoid health risks caused by insufficient or excess Se^(5,6)^. In spite of the data of daily Se intakes in Japanese adults^(7–9)^, little is known about Se intakes in Japanese during their early childhood. Generally, urinary Se excretion closely correlates with Se intake^(10,11)^. Therefore, we can estimate the Se intake in early childhood from the Se concentration and volume in and of 24-h urine. However, research on the intake of Se in early childhood is scarce due to the difficulty in collecting reliable 24-h urine samples from children and to the difficulty in quantifying urinary Se concentrations in children, which is lower than that in adults.

To overcome the first difficulty, we collected samples from free-living healthy Japanese children based on strict quality control^(12)^ and obtained written approval of the guardians of donor children for the secondary use of these samples for the present study.

To overcome the second problem, we developed a new measurement method to accurately evaluate Se concentrations directly in urine samples.

The objective of this study was to evaluate the daily Se intake by 3- to 5-year-old Japanese children. For this purpose, we developed an instrumental neutron activation analysis (INAA) method using the short-lived ^77m^Se (*t*_1/2_ = 17·4 s) nuclide for urinary samples, and measured Se in previously collected 24-h urine samples of 3- to 5-year-old children without any chemical separation and with high rapidity, precision and accuracy.

## Subjects and methods

### Subjects

Dietitians from Higashi-Matsushima (H) and Tome (T) city offices in Miyagi prefecture recruited 136 young children 2–5 years of age at their official health checks in October, November and December 2005 (for details, see Haga & Sakata^(12,13)^). Of these, seventy-nine subjects successfully provided 24-h urine samples, in which the successful collection was judged from the guardian's record (described as follows). Among them, fifty-three subjects 3–5 years of age consented to the additional use of seventy-two urine samples; the samples were kept frozen in our laboratory for the measurement of Se concentrations. We maintained the privacy of the children and their families by strict coding; i.e. only the involved dietitians of the city government kept the key information linking the data and individual identification.

### Urine sample origin

The study was conducted according to the guidelines of the Declaration of Helsinki. We used urine samples collected for our previously reported study^(12,13)^ after obtaining written informed consent from the guardians of the young subjects (*n* 53) according to the approval provided by the Ethical Committees of the National Institute of Health and Nutrition (approval no. 04029) and Shokei Gakuin University (approval no. 081218), Japan.

### Urine sample collection

We used the urine samples collected for our previous study^(12,13)^. The procedure of urine collection was based on Okubo's method^(14)^ with minor modifications, which was validated in our previous study^(12)^. Briefly, prior to the collection of the urine, parents of the subjects were instructed on how to collect the 24-h urine samples from their children at home or outside, and we requested the parents to perform definite recording. The parents of the children were asked to discard the overnight urine at 07.00 hours on day 1. Then, all the urine sample (from 07.00 to 07.00 hours on day 2) was collected, pooled, and stored in a cool (4°C) and dark place. The children were allowed to adhere a seal printed with fancy figures when they succeeded in collecting urine to encourage their motivation to cooperate and thereby minimise the failure in sample collection. The parents recorded the time of urination together with unsuccessful collection, fever, drug intake and diarrhoea on a record sheet. We used only urine samples with no such notation. Urine samples thus obtained were brought to the laboratory while being maintained in cool and dark conditions on the morning of day 2. After measuring the urine volumes, samples were aliquoted into plastic centrifuge tubes with lids (10 ml; Sanplatec) and frozen (−40°C) until analysis.

### Sample preparation for instrumental neutron activation analysis measurement

Several analytical techniques have been used in the past for the determination of Se in biological fluids including urine. These techniques include fluorometry, polarography, flame atomic absorption spectrometry (AAS), derivative AAS, electrothermal AAS, hydride generation AAS, derivative hydride AAS, inductively coupled plasma (ICP) atomic emission spectrometry, ICP-MS, GC-MS, liquid chromatography ICP-MS, HPLC-ICP-MS, X-ray fluorescence and neutron activation analysis (NAA)^(15–26)^. Various types of NAA techniques such as INAA, preconcentration NAA and radiochemical NAA have also been employed for this purpose^(27–30)^. The levels of Se in biological fluids are too low for direct determination by most instrumental analytical techniques. The task is further complicated by the presence of high concentrations of interfering elements such as Cl, Mg and Na in urine. Chemical separations are generally carried out to eliminate interferences as well as to improve detection limits. However, chemical separations are time-consuming and species dependent. Since all the species of a given element in urine samples being analysed are not known with great certainty, any given separation method may not be that reliable. Even with exhaustive precautions taken, chemical methods can contaminate samples, introduce high reagent blanks, and give low as well as irreproducible recovery of the element of interest, thereby adversely affecting the precision and accuracy of its measurement. It is therefore advisable to employ techniques that require minimum chemical manipulations of the sample. The previously frozen urine samples kept in our laboratory were thawed prior to analysis. A quantity of 1 ml of each sample was transferred to a pre-cleaned polyethylene irradiation vial using a calibrated Eppendorf pipette. These vials were pre-cleaned using the following steps: soaking overnight in 2 ml of 4 mol/l HNO_3_, washing them with tap water followed by distilled water, and drying them in an oven. A quantity of 0·7 g of pure sucrose was added to each vial containing 1 ml urine and dried overnight under an IR lamp in a fume hood. The vials were then heat-sealed.

### Selenium comparator standards

Se comparator standards were used in the present study for calculating Se concentrations in urine samples using the comparator NAA method. These standards were prepared from the plasma emission spectroscopy standard solution with a certified purity of >99·999 % supplied by SCP Canada Ltd. A quantity of 1 ml of the Se standard solution containing 0·2, 0·5 or 1·0 µg of Se was added to 0·7 g of sucrose in pre-cleaned polyethylene vials using the same procedure as the urine samples, capped and heat-sealed. As mentioned above, the Eppendorf pipettes were carefully calibrated prior to use for dilutions and transfers. The comparator standards were of identical geometry and contained approximately similar amounts of Se as the samples. The water used was first distilled in a quartz apparatus and then deionised using an ultrapure deionisation column. This distilled deionised water was used for making and diluting solutions and washing all apparatus. All materials and reagents used in the present study were analysed for ‘blanks' using experimental conditions similar to those of samples.

### Reference material

The INAA method was validated using a standard reference material (SRM) from the United States National Institute of Standards and Technology (NIST), namely NIST SRM 2670a Toxic Elements in Urine (Freeze Dried).

### Irradiation and instrumental neutron activation analysis counting

Samples, comparator standards and the reference material were irradiated for 10 s in a neutron flux of 5 × 10^11^/cm^2^ per s at the Ghana Research Reactor-1 (GHARR-1) facility in Accra, Ghana. After a 10-s decay time, the samples were counted for 30 s using a conventional γ-ray spectrometer. Up to four cycles of irradiation-decay counting were carried out in a pseudo-cyclic INAA (PC-INAA) method to explore if it would provide better results than the conventional INAA method. The Se levels were assayed using the 162-keV γ-ray of ^77m^Se.

### Estimation of daily selenium intake

In this study, we estimated the Se concentrations in early childhood using numerical values of the urinary Se excretion rate in Japanese adults. That is, we assumed that 73 % (male) and 77 % (female) of dietary Se were excreted into urine, based on results of a previous cohort study of the Japanese adult population^(7)^. The 24-h urinary Se excretion values were calculated as the product of the Se concentration in the urine samples and the volume of 24-h urine^(12)^. We then estimated the dietary Se intake by dividing the 24-h urinary Se excretion by 0·73 (male) or 0·77 (female).

### Body weight measurement

Body weight was measured to the 0·1 kg on calibrated scales at the official physical examination for 3-year-old children (*n* 18) within 2 months prior to the urine collection. For 4- to 5-year-old children (*n* 35), body weight was also measured to 0·1 kg on calibrated scales in the laboratory at the next day of urine sampling (day 2).

### Calculations and statistics

Urine samples used in the present report were obtained from a published observational study in healthy children (*n* 53)^(12)^. The primary outcome measure of the study was the estimation of daily Se intake by children. Given the Se analysis, the desired sample size for the study could not be calculated *a priori* and is therefore based on similar published studies that used Se analyses on 24-h urine samples. In particular, a sample size in the range of twelve to thirty subjects has been shown to give sufficient statistical power to estimate daily Se intake based on 24-h urine samples^(10,31,32)^.

Non-normally distributed data were subjected to log-transformation before analysis. Arithmetic and geometric means were calculated for 24-h urinary Se excretion and for estimated daily Se intake for fifty-three subjects. We also estimated the distribution of usual Se intake and the ratio of inter-/intra-individual variance in a population based on daily urinary Se excretion of the same individual on plural days employing the best-power method, in which the distribution was transformed to nearly normal and was adjusted for within-person variability. The best-power method was proposed by Nusser *et al*.^(33)^ and details of the method have been mentioned elsewhere^(33,34)^. For estimating the distribution of usual Se intake and the ratio of inter-individual:intra-individual variance, we used online software provided by Japan's National Institute of Public Health (Estimation software for the usual intake distribution version 1.2; https://www.niph.go.jp/soshiki/gijutsu/download/habitdist/habitdist.zip). We conducted a two-way ANOVA involving sex, location, age and their interaction effects using Bell Curve for Excel (Social Survey Research Information Co., Ltd) on a Windows computer. Differences between means were considered statistically significant at *P* < 0·05.

## Results

**Fig. 1.**
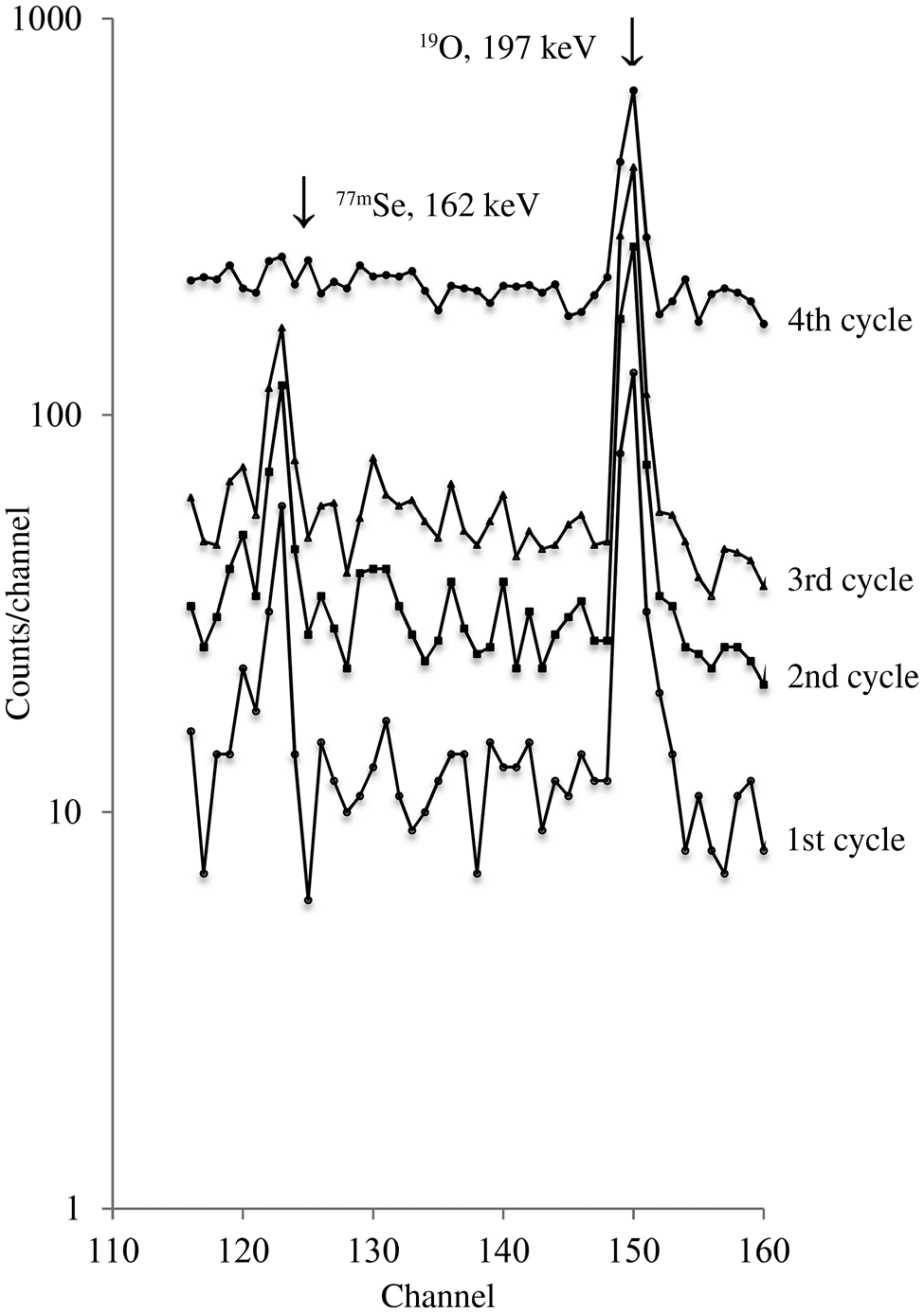
γ-Ray spectra of United States National Institute of Standards and Technology (NIST) Toxic Elements in Urine (SRM-2678) by pseudo-cyclic instrumental neutron activation analysis (PC-INAA).

In order to determine the number of cycles needed for optimum measurements, several samples of the NIST SRM 2670a Toxic Elements in Urine were analysed by the PC-INAA method for Se. The following concentrations in μg/l (cycle no.) were obtained: 201 (sd 65) (no. 1), 243 (sd 32) (no. 2), 207 (sd 31) (no. 3) and 211 (sd 28) (no. 4). Obviously, the precision of measurement improved with the increasing number of cycles without any noticeable effect on the central value of the measurement. The partial γ-ray spectra of this SRM for four cycles are shown in Fig. 1. It is evident that perhaps a comparably good result could be obtained from the first cycle. As the number of cycles increased, so did the background activity. Consequently, the Se levels in urine samples of the present study were measured using the first cycle. It is evident that this INAA method can be completed within 60 s of experimental time.

The INAA method was validated by analysing the NIST SRM 2670a Toxic Elements in Urine. The average of triplicate analyses was 229 (sd 23) µg/l compared with the certified value of 229·5 (sd 8·3) µg/l. A detection limit of 30·7 µg/l was obtained for this sample. Out of the seventy-two urine samples analysed by INAA (described as follows), only four samples were below the detection limit of 10 µg/l. When the Se levels in urine samples were below the detection limit, we arbitrarily equated its amount to 10 µg/l.

The 162-keV γ-ray of ^77m^Se is highly specific. It could be interfered with by the 162-keV γ-ray of ^116m2^In (*t*_1/2_ = 2·18 s) and 161-keV γ-ray of ^179m1^Hf (*t*_1/2_ = 18·7 s). Although no published report of In and Hf in human infant and toddler urine samples can be found in the literature, the half-life of ^77m^Se through its 162-keV γ-ray in several urine samples was measured and found to vary between 17·3 and 17·5 s, comparable with the literature value of 17·4 s, ruling out any interference. Moreover, the Se content of NIST SRM 2670a measured in the present study agree very well with the certified value, proving that the 162-keV γ-ray of ^77m^Se can be relied upon under the experimental conditions used. It can also be inferred that there was no Se reagent blank from sucrose as well as no loss of Se during drying under an IR lamp in the sample preparation steps.

It can be concluded from the above discussions that the INAA method for the determination of Se in urine samples does not require any chemical separation and can be accomplished with high precision, accuracy and rapidity. This method was then used to analyse seventy-two urine samples.

**Fig. 2.**
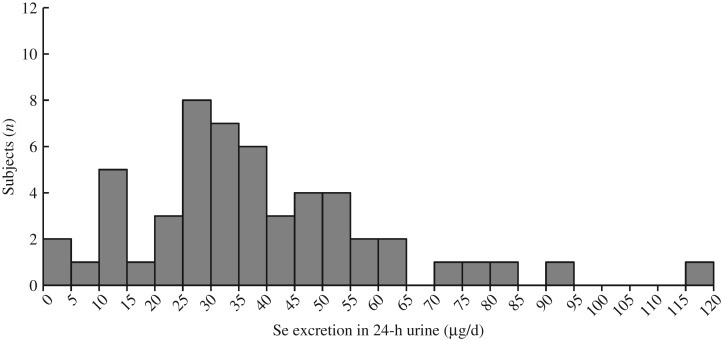
Histogram for distribution of 24-h urinary selenium excretion (*n* 53).

The distribution of Se concentrations in the fifty-three 24-h urine samples, excluding 2nd and 3rd day samples, was log-normal, skewed to the high concentration side (Fig. 2). Therefore, the data were log-transformed before statistical analysis.

**Fig. 3.**
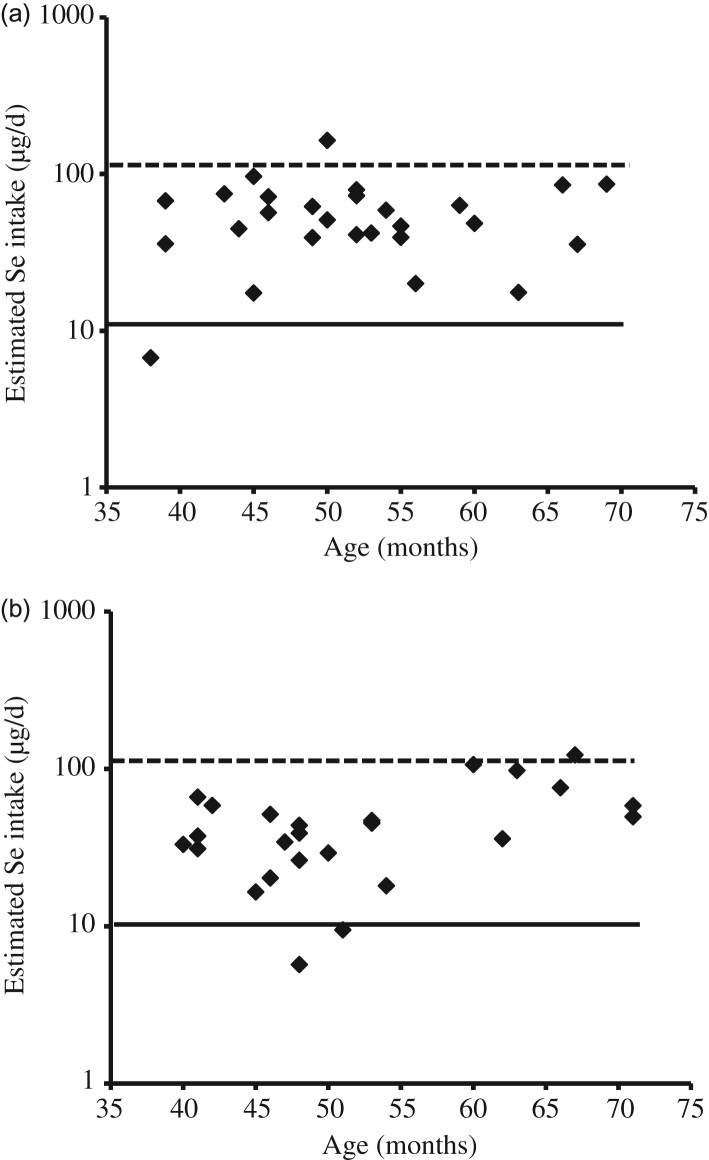
Estimated selenium intake levels in (a) male (*n* 27) and (b) female (*n* 26) children of 3–5 years of age in Higashi-Matsushima and Tome cities, Miyagi prefecture, Japan. Solid and broken horizontal lines show the recommended dietary allowance and tolerable upper intake level of selenium from the Dietary Reference Intakes for Japanese (2015)^(35)^, respectively.

Collection of 24-h urine was conducted only once in T city and more than once (three times from eight subjects and twice from three subjects) in H city. Therefore, urine data only from the first successful collection were included to test effects of city, sex and age. The age effect on body weight, daily urinary Se excretion and daily Se intake was statistically significant (Table 1). The mean estimated Se intake of the fifty-three subjects was 51·5 µg/d (geometric mean: 42·7 µg/d) (Table 1). Estimated Se intake level of twenty-seven male and twenty-six female subjects plotted against their age (Fig. 3) shows that the Se intakes in one male subject, with 6·7 µg/d, and two female subjects, with 5·7 and 9·5 µg/d, respectively, were below Japanese recommended dietary allowance of Se in children aged 3–5 years (10 and 15 µg/d in female and male subjects, respectively)^(35)^. In addition, one male subject (164·2 µg/d) and one female subject (122·6 µg/d) exceeded the tolerable upper intake level of Se in children aged 3–5 years (110 µg/d for both sexes)^(35)^.

**Table 1.** Urinary selenium concentration, urinary selenium excretion and estimated selenium intake in healthy 3- to 5-year-old children who succeeded in providing complete 24-h urine in Miyagi prefecture, Japan† (Mean values and standard deviations; geometric means)

		Age (months)		Body weight (kg)		Volume of 24-h urine‡ (ml/d)		Se concentration in 24-h urine (μg/l)		Se excretion in 24-h urine (μg/d)			Se excretion in 24-h urine (μg/kg body weight/d)			Estimated daily Se intake§ (μg/d)		
	Subjects (*n*)	Mean	sd	Mean	sd	Mean	sd	Mean	sd	Mean	sd	Geometric mean	Mean	sd	Geometric mean	Mean	sd	Geometric mean
All subjects	53	51·9	8·9	17·5	2·9	515·3	167·8	79·4	47·8	38·5	22·5	32·0	2·2	1·3	1·9	51·5	30·2	42·7
Sex																		
Male	27	51·7	8·4	17·1	1·8	535·3	186·2	82·5	49·0	41·2	22·9	34·9	2·4	1·5	2·1	56·5	31·4	47·9
Female	26	52·1	9·5	17·9	3·7	494·6	147·1	76·0	47·2	35·7	22·1	29·2	1·9	1·0	1·6	46·3	28·7	37·9
Location																		
T city	37	53·3	8·9	17·8	2·9	534·9	184·1	80·9	46·8	40·2	22·9	34·2	2·3	1·3	1·9	54·0	31·0	45·9
H city	16	48·8	8·4	16·4	2·6	470·0	114·7	75·8	51·3	34·7	21·6	27·4	2·1	1·1	1·6	45·8	28·4	36·2
Age (months)																		
36–53	35	46·7	4·5	16·3	1·9	509·1	176·8	72·9	40·4	35·4	21·4	29·2	2·2	1·4	1·8	47·5	29·5	38·9
54–71	18	62·1	5·8	19·9*	3·1	527·5	152·8	92·0	58·9	44·5*	23·8	38·2	2·2	1·1	1·9	59·2*	30·9	51·0

H, Higashi-Matsushima; T, Tome.

* Statistically significant with ANOVA (*P* < 0·05, *v.* 36–53 months).

† All one-way effects and two-way interaction effects were statistically not significant except for age effect on body weight, daily urinary Se excretion and Se intake.

‡ Values in our previous report^(12)^.

§ (Urinary Se excretion)/(0·73 (male) or 0·77 (female)).

We used data from two and eight subjects (three male and seven female) among sixteen subjects from H city who successfully collected 24-h urine over two and three consecutive days, respectively, to estimate the distribution of usual daily Se intake by the best-power method^(33)^. Thus estimated usual Se intake was 37·4 (sd 5·9) µg/d. Based on the logarithmically transformed data of subjects, the geometric mean was 27·7 µg/d (Table 2) and the ratio of intra-individual:inter-individual variances was 16·7 (28·0/1·7). The 5th to 95th percentile of usual Se intake of these subjects was 17·5 to 40·4 µg/d (Table 2), which ranged between the recommended dietary allowance and tolerable upper intake level of Se by the Dietary Reference Intakes for Japanese (2015)^(35)^.

**Table 2.** Distribution of usual urinary excretions of selenium and usual selenium intake in ten subjects

Percentile	Usual urinary excretions of Se* (μg/d)	Estimated usual Se intake (μg/d)	Usual Se intake* (μg/d)
5th	13·1	26·6	17·5
10th	13·1	27·4	18·2
25th	18·5	34·8	25·6
50th	19·5	36·4	27·1
75th	24·2	40·1	31·2
90th	30·1	48·6	39·4
95th	30·9	49·6	40·4
Mean	–	37·4	–
sd	–	5·9	–
Geometric mean	21·2	–	27·7
Ratio of intra- individual:inter individual variances	14·5 (23·2/1·6)	19·1 (1·54/0·08)	16·7 (28·0/1·7)

* Estimated from logarithmically transformed data.

## Discussion

The present observational study evaluated the daily Se intake of 3- to 5-year-old Japanese children. In addition, the distribution of usual dairy Se intake and the ratio of inter-/intra-individual variance were estimated. To the best of our knowledge, this is the first study to establish the daily Se intake by 3- to 5-year-old Japanese children based on Se excretion in 24-h urine samples.

Tsuda *et al*.^(36)^ reported the urinary concentrations of Se in healthy Japanese children aged 0–4 and 5–9 years (34·9 and 35·2 µg/l in males and 32·6 and 44·2 µg/l in females, respectively). Their reported values were smaller than those of the 3- to 5-year-olds in the present study (82·5 in males and 76·0 µg/l in females; Table 1). Tsuda *et al*.^(36)^ measured Se concentrations of spot urine samples by a fluorometric method using 2,3-diaminonaphthalene. Thus, the differences in sample source (spot *v.* 24-h urine) and in analytical method might be responsible for the difference. The daily urinary Se excretions of Japanese children aged 3–5 years old in the present study (Table 1) were about one-quarter of those in adult males and females (geometric mean: 129·2 and 108·3 µg/d, respectively)^(7)^. The dietary sources of Se were not revealed in the present study. Zhou *et al*.^(37)^ and Yamashita *et al*.^(38)^ reported that seafood is one of the possible source of Se in Japan, while Abdulah *et al*.^(39)^ reported low contributions of rice and vegetables to the daily intake of Se in Japan.

The body weight and daily urinary Se excretion of 54- to 71-month-old children were significantly higher than those of 36- to 53-month-old children, whereas the daily urinary Se excretion per kg body weight was statistically not significant (Table 1). Thus, the children aged 54–71 months, who were heavier in body weight and excreted more urinary Se than children aged 36–53 months, excreted the same amount of urinary Se per kg body weight. Therefore, we speculated that in 3- to 5-year-old children, daily urinary Se excretion might have increased proportionally to body growth. Likewise, the estimated daily Se intake of 54- to 71-month-olds was significantly higher than those of 36- to 53-month-olds (*P* < 0·05).

For measurement of low levels of Se in the 24-h urine samples, we developed a new INAA method. Se has six stable isotopes which can produce seven radionuclides on thermal neutron activation. However, the most suitable radionuclides for measurement are ^75^Se and ^77m^Se. The ^75^Se nuclide is long-lived (*t*_1/2_ = 118·5 d); it requires lengthy irradiations at a high neutron flux to produce sufficient activity for small amounts of Se, long decay to reduce interferences from major elements in urine, and long counting periods to accumulate statistically significant number of counts. The total experimental time is at least 2 weeks, which can increase the cost of analysis and also make routine analysis for Se in urine a lengthy process. Alternatively, the short-lived ^77m^Se nuclide (*t*_1/2_ = 17·4 s) can be routinely used^(40–44)^ for measuring Se levels. The conventional INAA procedure involves irradiation, decay and counting of a sample. These three steps can be repeated immediately one after the other to improve precision and detection limit in a technique appropriately called cyclic INAA (CINAA). If several minutes to days are allowed to elapse between repetitions of these cycles, then the technique is called pseudo-cyclic INAA (PC-INAA). In the past, several CINAA and PC-INAA methods were developed in our laboratory for the determination of Se in various matrices^(40–47)^ but not urine. In the study reported here, the short-lived nuclide ^77m^Se was applied for the first time, to the best of the authors’ knowledge, to the direct determination of Se in urine samples without any chemical separation.

One limitation of the present study was that the estimated Se intake was calculated based on the proportion of urinary Se excretion in oral Se intake obtained from Japanese adults aged between 40 and 59 years. Although we are not entirely sure if the proportion also stands for young children, the value of urinary Se excretion per kg body weight in our study (geometric mean: 2·1 and 1·6 µg/kg per d in male and female children, respectively) (Table 1) was close to the reported value of the Japanese adult (1·9 and 2·0 µg/kg/d, respectively)^(7)^. Our results are also consistent with that in the adult there was no sex difference in the value of urinary Se excretion per body weight^(7)^. On the other hand, indices reflecting the Se status in the blood (such as plasma Se concentration or glutathione peroxidase activity) decrease in normal term infants during the first several months of age and then steadily increase during later infancy and early childhood to reach a plateau at approximately 20 years of age^(48)^. It might be reflecting that the proportion of Se in dietary intake that accumulates within the body is higher in early childhood than in adults. Therefore, for early childhood, it might be necessary to add the quantity of Se that accumulates in the body in connection with growth when we estimate oral intake of Se from urinary excretion of Se. Thus, there is a possibility that we have underestimated Se intake in the present study.

In addition, it would be premature to extrapolate our findings only from two cities in a prefecture to Japanese children in general. However, there have been no reports on the daily Se intake by 3- to 5-year-old Japanese children. Therefore, these findings will be useful for establishing early childhood Se intake standards in Japan.

### Conclusions

We evaluated the daily Se intake of 3- to 5-year-old Japanese children based on Se excretion in 24-h urine samples quantified by a new INAA method. Our data suggested that Se intake of children from two different cities localised in Japan was adequate. In future, the INAA method for urinary samples can be used for the determination of Se status of various groups of subjects not only in Japan but in the world.
